# An exploratory study of client and provider experience and perceptions of facility-based childbirth care in Quiché, Guatemala

**DOI:** 10.1186/s12913-022-07686-z

**Published:** 2022-05-03

**Authors:** Reena Sethi, Kathleen Hill, Suzanne Stalls, Susan Moffson, Sandra Saenz de Tejada, Leonel Gomez, Miguel Angel Marroquin

**Affiliations:** 1 Jhpiego, Baltimore, USA; 2 Momentum Country and Global Leadership, Washington, USA; 3 Independent Researcher, Guatemala City, Guatemala; 4 Reproductive Health Independent Researcher/Consultant, Guatemala City, Guatemala; 5 Jhpiego, Guatemala City, Guatemala

**Keywords:** RMC, Humanizing childbirth, Mistreatment, Human rights abuse, Obstetric violence, Experience of care, Professional-patient relationship, Quality of care, Childbirth, Guatemala

## Abstract

**Introduction:**

Respectful maternity care (RMC) is fundamental to women’s and families’ experience of care and their decision about where to give birth. Studies from multiple countries describe the mistreatment of women during facility-based childbirth, though only a small number of studies from Guatemala have been published. Less information is available on women’s negative and positive experiences of childbirth care and health workers’ perceptions and experiences of providing maternity care.

**Methods:**

As part of a program implemented in the Western Highlands of Guatemala to improve quality of reproductive maternal newborn and child health care, a mixed methods assessment was conducted in three hospitals and surrounding areas to understand women’s and health workers’ experience and perceptions of maternity care. The quantitative component included a survey of 31 maternity health workers and 140 women who had recently given birth in these hospitals. The qualitative component included in-depth interviews (IDIs) and focus group discussions (FGDs) with women and maternity health workers and managers.

**Results:**

Women reported a mix of positive and negative experiences of childbirth care related to interpersonal and health system factors. 81% of surveyed women reported that health workers had treated them with respect while 21.4% of women reported verbal abuse. Fifty-five percent and 12% of women, respectively, reported not having access to a private toilet and bath or shower. During IDIs and FGDs, many women described higher rates of verbal abuse directed at women who do not speak Spanish. A regression analysis of survey results indicated that speaking Ixil or K’iche at home was associated with a higher likelihood of women being treated negatively during childbirth in a facility. Health worker survey results corroborated negative aspects of care described by women and also reported mistreatment of health workers by clients and families (70.9%) and colleagues (48.2%).

**Conclusions:**

This study adds to the literature on women’s experience of institutional childbirth and factors that influence this experience by triangulating experience and perceptions of both women and health workers. This assessment highlights opportunities to address mistreatment of both women and health workers and to build on positive care attributes to strengthen RMC for all women.

## Background

Person-centered care is an essential element of quality care that deeply affects women’s, newborns’ and families’ experience of childbirth and their decisions about where to give birth. A growing body of literature from around the world describes the quality of facility-based childbirth care, including the effectiveness, safety and person-centeredness of care with respect to normative standards and women’s experience of care. A systematic qualitative review of what matters to women in childbirth found that most women want a positive experience that fulfils or exceeds their personal and sociocultural beliefs and expectations, including “giving birth to a healthy baby in a clinically and psychologically safe environment with practical and emotional support from birth companions, and competent, reassuring, kind clinical staff” [1]. The 2015 World Health Organization (WHO) Quality of Care vision for maternal and newborn health outlines eight aspirational quality standards, including provision of evidence-based (clinical) care, essential health system functions (referrals, information systems, commodities, human resources) and women’s and newborns’ experience of care categorized within three domains: effective communication, respect and dignity, and emotional support [2].

Assessments of the quality of clinical and person-centered respectful maternity care (RMC) from around the world demonstrate significant deficits based on women’s self-reported experience of care and observed (or documented) adherence with normative standards, including the outright mistreatment of many women. Despite the many published studies assessing RMC and mistreatment of women during facility-based childbirth [3–5], there is sparse evidence available to inform and guide the design, routine monitoring and implementation of approaches to promote RMC and reduce mistreatment in comprehensive maternal and newborn health programs. In addition, there is growing awareness of the difficulties faced by maternity health care workers in many settings, including the mistreatment of health workers [6]. Experience assessing and regularly monitoring women’s experience of childbirth care, health workers’ experience of providing maternity care and health system factors to inform the local design and monitoring of program interventions to promote RMC and reduce mistreatment remains limited. Many recent studies on RMC, including women’s reported experience of childbirth care, are from sub-Saharan Africa and Southeast Asia, with fewer published studies from the Latin America and Caribbean (LAC) region, though there is a rich and long history of advocacy and policy work in the LAC region to “humanize childbirth” and reduce inequities in birth outcomes for marginalized women, including indigenous women. Only a small number of studies have examined women’s experience and perceptions of the quality of care during childbirth in facilities or the experience of indigenous people in health care facilities in Guatemala, the site of this study [7–11].

Despite encouraging progress, Guatemala’s maternal mortality ratio (MMR) of 108 deaths per 100,000 live births is among the highest in Central America [12] and the estimated MMR among indigenous women of Mayan descent in Guatemala is 1.75 times higher than among non-indigenous women (referred to as *Ladinas.*) [13]. The indigenous population in Guatemala (about half of the population) has been socially and economically disadvantaged for centuries and the majority of Mayan Guatemalans reside in the Western Highlands region, the geographic area of this study [13]. In Quiché, one of the departments in the Western Highlands, 34% of the women give birth in facilities as compared to a national average of 65% [14]. Several factors contribute to the lower utilization of facility birth services among indigenous women including: sociocultural values and customs associated with a preference for a home birth with a traditional birth attendant (*comadrona);* geographic obstacles to accessing care among indigenous women living in isolated rural areas; language barriers and, potentially, poorer quality of interpersonal and clinical care for indigenous women in public facilities [15]. In response to these factors, in 2011, the Ministry of Public Health and Social Assistance (MSPAS) issued guidelines for “pertinencia cultural” (cultural relevance) to promote culturally sensitive care and to bridge gaps between the biomedical system and an indigenous community which holds strong beliefs in a system of “ancestral medicine” that has guided health behaviors for centuries [16].

From 2016 to 2019 the global Maternal and Child Survival Program (MCSP), funded by United States Agency for International Development (USAID), collaborated with country counterparts in five departments in the Western Highlands of Guatemala to improve the quality of reproductive, maternal, newborn, child and adolescent health care and nutrition services, including person-centered and culturally responsive childbirth services. To support the local design and implementation of program interventions to promote RMC and reduce mistreatment in MSPAS facility childbirth services in the Western Highlands, the project conducted a mixed-method assessment in 2018 of women’s perceptions and experience of childbirth care and of health workers’ experience of providing care in three hospitals and surrounding communities in the Quiché department. MCSP and MSPAS then convened community and hospital stakeholders to analyze assessment findings, discuss drivers of respectful care and of mistreatment, and to design local interventions to improve RMC and reduce mistreatment.

## Methods

### Study design and setting

This was a mixed-methods quantitative and qualitative assessment in three hospitals and surrounding communities in three municipalities (districts) in the Quiché department in the Western Highlands (Nebaj, Uspantán and Santa Cruz). Qualitative methods included community-based focus group discussions (FGDs) and in-depth interviews (IDIs) with women of reproductive age, community members, *comadronas*, as well as facility-based interviews with hospital administrators and maternity health care workers. Quantitative methods included a survey of women who had recently given birth in the three hospitals and a survey of health workers providing maternity care services in the selected facilities. Qualitative and quantitative methods examined stakeholders’ perspectives, experience of, and observations of RMC and mistreatment of women in childbirth as well as health worker experience of providing childbirth care in study facilities.

### Study recruitment and sampling

Three hospitals and surrounding communities in the Quiche directorate were purposively selected to participate in the assessment based on a high birth volume and serving a high proportion of indigenous Guatemalans. In the Quiche directorate, there are approximately 23 public and private facilities that provide childbirth services. These include 4 hospitals that provide referral-level childbirth services (3 of which are the hospitals in this study), in addition to a few private facilities as well as 16 centers of permanent care or CAPs (*Centros de Atención Permanente*) which are primary care facilities where women may access childbirth services 24 hours, 7 days a week. Women with complications are referred to hospitals. A total of 102 qualitative interviews were conducted, including 54 community-based interviews with women of reproductive age who had previously delivered in a facility or at home, community–based interviews with six *comadronas*, and facility-based interviews with 12 health administrators and facility maternity care providers, and 9 other facility stakeholders including community liaison coordinators and social workers. Six community-based FGDs (5–8 participants each) were conducted with women of reproductive age, including both users and non-users of hospital maternity care, in 2 villages in each of the 3 hospital municipalities. The qualitative sample was a purposeful sample that was determined by the project resources and timeline. The FGDs and IDIs focused on experience of care and perceptions of RMC and mistreatment and were adapted from published qualitative tools used in the formative assessment phase of a four-country assessment of mistreatment prevalence [4]. A total of 171 quantitative surveys were conducted, including 31 surveys of maternity health care workers working in the three hospitals and 140 surveys of women who had recently given birth in these health facilities. The client survey tools were adapted and expanded based on client survey tools from published studies [17, 18], including a Person-Centered Maternity Care (PCMC) scale validated in Kenya, Ghana and India [5]. The provider survey tool was developed for the purpose of this study and assessed providers’ attitudes and experience of working in the hospitals, as well as provider observations of how women are treated in childbirth in their hospital and their observations of how health care worker colleagues are treated by clients, families and other colleagues. The provider survey adapted several items from the Maslach Burnout Inventory (MBI), an instrument that has been extensively validated and used to assess occupational burnout including among health workers [19].

### Study procedures

MCSP program staff made a courtesy visit to introduce the planned data collection activities and obtain permission to conduct the assessment from the Vice Ministry of Hospitals, the municipal MSPAS authorities, and directors of the three hospitals, and of the maternity in each hospital. At the community level, the team met with community health workers *(madres gu*í*as*) to request their assistance to identify *comadronas* and women who had given birth at home or in a facility within the prior two years, who might be willing to participate in IDIs and FGDs. Together, the data collector and *madre guia* visited the homes of women who agreed to an interview, where the interview was conducted by a member of the study team after obtaining consent. The *madre guía* also helped to arrange IDIs with *comadronas* who agreed to participate. Women were asked their language preference for the interviews (Spanish, Kiche and Ixil) and interviews were conducted in the woman’s language of choice by a female native Kiche, Ixil or Spanish speaker. Most women chose to be interviewed in Ixil or Kiche. All IDIs and FGDs were recorded, those in Spanish transcribed verbatim, and those in the Mayan languages were translated into Spanish.

In each of the three hospitals and with the help of the maternity head nurse, a member of the study team approached recently delivered/postpartum women to request their permission to participate in the survey. After obtaining informed consent, survey questionnaires were administered in a private cubicle area in two of the hospitals and at the bedside of the third hospital (where no private area was available). In each hospital, the maternity head nurse identified a minimum of ten maternity care providers, including nurses with several years of experience and direct contact with clients, an obstetrician-gynecologist, and the maternity head, to request their participation in the survey. After giving consent, providers who agreed to participate completed the survey questionnaire independently. Facility health worker individual interviews were conducted in a private area after obtaining consent and permission to record the interview.

### Data analysis

Qualitative Analysis: The interviews and FGDs were recorded, translated and transcribed almost in their entirety (paraphrasing was used about 10% of the time). Transcripts were coded using MaxQDA software, using both predefined codes related to the questions asked, and emergent codes developed from the analyzed data. Data from interviews and FGDs were divided into four groups according to the interviewing technique and the type of interviewee. A single qualitatively trained researcher coded all of the qualitative data.

Quantitative Analysis: For the client exit survey, data from the survey forms were input into CSPro 6.3. Data were analyzed using Stata versions 14 and 15 (StataCorp LLP, College Station, TX, USA). A score was then developed for positive aspects of care (total number of ‘yes’ responses divided by the total number of questions related to positive care [*n* = 14]) and for negative aspects of care (total number of ‘yes’ responses divided by the total number of questions related to negative care [*n* = 10]) listed in Table 2. Bivariate Poisson regression models were used to examine the difference in scores based on the background characteristics of the client including woman’s age category, marital status, parity, language spoken at home, place of residence, residence in urban or rural location, highest education and socioeconomic status. Significance was set at *P* < 0.05 and for *p* < 0.10 for inclusion into the multivariate model.

## Results

Table 1 shows the sociodemographic characteristics of the women who completed the survey in the 3 study hospitals as well as the characteristics of maternity care health workers. The mean age of the 140 surveyed women was 24.9 (SD: 6.2) years old and more than half (55.7%) had attended primary school. Their mean number of births was 2.2 (SD: 1.6). Nearly one fifth of women (17.8%) on average across the three hospitals reported speaking Ixil at home and 60% of women reported speaking K’iche’ at home. Approximately one fifth of women on average reported to speak Spanish at home, ranging from 11.1% to 22.2% across the three hospitals. More than half of surveyed women reported having a cesarean section (ranging from 67.7% in Hospital #2 to 41.7% of surveyed women in Hospital #1 to 30.6% of women in Hospital #3 (data not shown). Approximately three quarters of the 31 surveyed health workers from the maternities in the three hospitals were nurses (77%) with an average age of 34 years. Two thirds of surveyed health workers were women (67.7%).

**Table 1:** Characteristics of women and heath care workers participating in the survey in the three hospitals

Sociodemographic characteristics of women surveyed	Hospital #1	Hospital #2	Hospital #3	Total
	*N* = 36 (%)	*N* = 68 (%)	*N* = 36 (%)	*N* = 140 (%)
Age (mean, SD)	24.0 (6.7)	25.6 (6.3)	24.5 (5.4)	24.9 (6.2)
Age category				
15–19	9 (25.0)	13 (19.1)	8 (22.2)	30 (21.4)
20–24	15 (41.7)	23 (33.8)	12 (33.3)	50 (35.7)
25–29	3 (8.3)	14 (20.6)	11 (30.6)	28 (20.0)
30–34	4 (11.1)	10 (14.7)	2 (5.6)	16 (11.4)
35 +	5 (13.9)	8 (11.8)	3 (8.3)	16 (11.4)
Highest level of schooling attended				
None	2 (5.6)	4 (5.9)	8 (22.2)	14 (10.0)
Primary	21 (58.3)	44 (64.7)	13 (36.1)	78 (55.7)
Secondary +	13 (36.1)	20 (29.4)	15 (41.7)	48 (33.3)
Language spoken at home				
Ixil	25 (69.4)	0 (0.0)	0 (0.0)	25 (17.8)
K’iche’	7 (19.4)	52 (76.5)	25 (69.4)	84 (60.0)
Spanish	4 (11.1)	2 (20.6)	8 (22.2)	26 (18.6)
Other	0 (0.0)	2 (2.9)	3 (8.3)	5 (3.6)
Socioeconomic index (household)^a^:				
Quintile 1	9 (25.0)	11 (16.2)	9 (25.0)	29 (20.7)
Quintile 2	4 (44.4)	26 (38.4)	9 (25.0)	39 (27.9)
Quintile 3	23 (63.9)	31 (45.6)	18 (50.0)	72 (51.3)
Quintile 4	0 (0.0)	0 (0.0)	0 (0.0)	0 (0.0)
Quintile 5	0 (0.0)	0 (0.0)	0 (0.0)	0 (0.0)
Number of births (mean, SD)	2.2 (1.5)	2.3 (1.7)	2.2 (1.4)	2.2 (1.6)
**Sociodemographic characteristics of health workers surveyed**				
	*N* = 10	*N* = 11	*N* = 10	*N* = 31
Age (mean, SD)	30.1 (7.0)	33.6 (8.2)	38.8 (10.0)	34.2 (8.9)
Gender = female (%)	5 (50.0)	10 (90.9)	6 (60.0)	21 (67.7)
**Occupation**				
Nurse	6 (60.0)	10 (90.9)	8 (80.0)	24 (77.4)
General practitioner	1 (10.0)	0 (0.0)	2 (20.0)	3 (9.7)
Gynecologist/obstetrician	3 (30.0)	1 (9.1)	0 (0.0)	4 (12.9)
**Services provided:**				
Family planning	4 (40.0)	3 (27.3)	5 (50.0)	12 (38.7)
Antenatal care	4 (40.0)	7 (63.6)	7 (70.0)	18 (58.1)
Childbirth	10 (100.0)	11 (100.0)	10 (100.0)	31 (100.0)
Postpartum care	6 (60.0)	8 (72.7)	4 (40.0)	18 (58.1)
**Weekly workload (hours)**				
Less than 40 (%)	0 (0.0)	3 (27.3)	1 (10.0)	4 (12.9)
40 and above (%)	10 (100.0)	8 (72.7)	9 (90.0)	27 (87.1)
**Training received** (%)				
Interpersonal skills	4 (40.0)	5 (45.5)	9(90.0)	18 (58.1)
Gender	3 (30.0)	1 (9.1)	6 (60.0)	12 (38.7)
Culturally sensitive care	4 (40.0)	4 (36.3)	6 (60.0)	14 (45.2)
Human rights	5 (50.0)	5 (45.4)	7 (70.0)	17 (54.8)
Respectful care	7 (70.0)	6 (54.5)	6 (60.0)	21 (67.7)

^a^Calculated using 6 indicators: (toilet, drinking water source, cooking fuel, roof material, wall material, and floor material) using weights from Equity Tool (https://www.equitytool.org/guatemala/), *SD* Standard Deviation

### Women’s experience of childbirth care

Table 2 presents results from the survey of 140 women who had recently given birth in the three study hospitals. The survey mean “positive care score” was 9.4 (SD: 3.2) out of 14 total items. The most common positive elements of childbirth care reported by postpartum women included “health workers spoke to me in a language that I speak” (94.3%) followed by “health workers took the best care of me they could” (85.0%) followed by “health workers did everything they could to make me more comfortable” (82.9%) and then “being treated with respect by health workers (80.7%). Conversely, the least commonly reported positive care elements included “health workers explaining what was happening and what to expect” (34.3%), “being allowed to have a desired companion during labor” (19.3%) and “being allowed to have a desired companion during delivery” (12.1%). One-half to three quarters of the 140 women surveyed in the three hospitals felt that providers were kind and friendly, ranging from 47% in Hospital #3 to 77.8% in Hospital #1. During community-based qualitative interviews, several women described positive experiences of facility childbirth care, mostly centered around being spoken to politely, feeling that personnel cared for her and looked after her, and explanations were given for procedures for herself or her baby.

*I was well treated by the nurses who cared for me. My husband could also tell they were treating me well and he thanked them too….they were very kind to me…they told me what I had to do an hour before my baby was born and explained it many times.* (IDI with a woman who had previously delivered in a facility, Nebaj)

**Table 2:** Client-reported RMC and mistreatment during childbirth in three hospitals

	Hospital #1	Hospital #2	Hospital #3	Total	*p*-value
	*N* = 36	*N* = 68	*N* = 36	*N* = 140	
	n(%)	n(%)	n(%)	n(%)	
**Respectful Care (Positive Care Attributes)**					
Was allowed to have desired companion in labor^a1^	25 (69.4)	1 (1.5)	1(2.8)	27 (19.3)	*p* = .001
Was allowed to have desired companion during delivery^a2^	16 (44.4)	0 (0.0)	1 (2.8)	17 (12.1)	*p* = .009
Was covered with a cloth or blanket so did not feel exposed^a^	23 (63.9)	49 (72.1)	14 (38.9)	86 (61.4)	*p* = .020
Health worker treated her with respect^a^	34 (94.4)	52 (76.5)	27 (75.0)	113 (80.7)	*p* = .052
Staff were kind and friendly	28 (77.8)	43 (63.2)	17 (47.2)	88 (62.9)	*p* = .255
Staff helped manage pain	30 (83.3)	49 (72.1)	31 (86.1)	110 (78.6)	*p* = .425
Health workers did everything they could to help her be more comfortable	32 (88.9)	53 (77.9)	31 (86.1)	116 (82.9)	*p* = .609
Health workers took the best care of her in the way that they could^a^	30 (83.3)	58 (85.3)	31 (86.1)	119 (85.0)	*p* = .978
Health workers paid attention if she asked for help^a^	29 (80.6)	36 (52.9)	25 (69.4)	90 (64.3)	*p* = .057
Health workers spoke to you in a language that you speak^a^	31 (86.1)	66 (97.1)	35 (97.2)	132 (94.3)	*P* = .617
Health workers provided explanations for exams or procedures or why they gave you medicine	18 (50.0)	39 (57.4)	27 (75.0)	84 (60.0)	*p* = .163
Health workers asked for consent before doing exams, procedures, or giving medicine^a^	15 (41.7)	55 (80.9)	29 (80.6)	99 (70.7)	*p* = .002
Health workers explained what was happening and what to expect^a^	25 (69.4)	10 (14.7)	13 (36.1)	48 (34.3)	*p* = .001
She felt she could ask questions about her care^a^	26 (72.2)	38 (55.9)	26 (72.2)	90 (64.3)	*p* < .001
Mean positive score (SD)	10.2 (2.5)	8.5 (3.2)	10.1 (3.5)	9.4 (3.2)	*p* = 0.817
**Mistreatment (Negative Care Attributes)**					
She was treated roughly physically by health providers^a^	1 (2.8)	1 (1.5)	0 (0.0)	2 (1.4)	*p* = .980
She experienced verbal abuse (shouting, insulting, scolding, being made fun of) ^a^	4 (11.1)	21 (30.9)	5 (13.9)	30 (21.4)	*p* = .169
She was restrained so she couldn’t move	1 (2.8)	0 (0.0)	0 (0.0)	1 (0.7)	*p* = .970
Health workers asked for money or informal payment for better care	0 (0.0)	0 (0.0)	0 (0.0)	0 (0.0)	*p* = 1.000
Downward pressure placed on her abdomen before baby was born	8 (22.2)	2 (2.9)	12 (33.3)	22 (15.7)	*p* = .029
Staff said she or her baby would not be healthy if she did not comply	2 (5.6)	4 (5.9)	2 (5.6)	8 (5.7)	*p* = .994
Health worker or staff made negative comments about her sexual activity	1 (2.8)	1 (1.5)	0 (0.0)	2 (1.4)	*p* = .980
She was sexually harassed or was touched inappropriately	0 (0.0)	0 (0.0)	0 (0.0)	0 (0.0)	*p* = 1.000
Health workers discussed her private information so that others could hear^a^	1 (2.8)	22 (32.5)	1 (2.8)	24 (17.1)	*p* = .010
She gave birth without a health worker helping her	1 (2.8)	1 (1.5)	2 (5.6)	4 (2.9)	*p* = .943
Mean negative score (SD)	0.72 (1.6)	0.98 (0.80)	0.64 (0.80)	0.83 (1.22)	*p* = .698

^a^Item from PCMC scale developed by Afulani and colleagues [5]

The survey mean “negative care score” was 0.83 (SD: 1.82) out of ten total mistreatment items. The most common forms of mistreatment reported by surveyed women were verbal abuse (21.4%) (scolding, shouting, insulting), discussing private information so that others could hear (17.1%), abdominal fundal pressure (15.4%), and threats that her baby would not be healthy if she did not comply (5.7%). Very few women or no women reported physical abuse (1.4%) or sexual abuse (0.0%). There were some differences in client reported RMC and mistreatment by hospital as shown in Table 2.

During community-based FGDs and IDIs, many women who had previously given birth in facilities described personally experiencing verbal abuse, while some women who had never given birth in a facility described hearing about verbal abuse from women who had given birth in a facility. Many women mentioned verbal abuse as a reason they don’t seek health care in health facilities:

*I don’t like how badly we’re treated [at the hospital]. They start telling us “if you didn’t feel pain when you were with your husband, why are you screaming here?” I don’t like the way they treat us, the horrible things they tell us, it’s just awful. Isn’t the nurse a woman too? As if they hadn’t suffered from this sort of pain to treat us like that. They treat us bad and that’s why I don’t want to go back to the hospital anymore. That’s why I labored at home and had my little boy with the midwife.* (IDI with a woman who had previously delivered in a facility and subsequently chose to deliver at home, Santa Cruz)

*They just tell you: “Take off what you have on and put on the gown, you know what you’re in for”. Some doctors treat you like an animal, they don’t care about your pain at the time. And when I complained I was told: “Okay, then why did you open your legs?” They said that in front of everyone, it was very humiliating.* (IDI with a woman who had previously delivered in a facility, Uspantan)

During both IDIs and FGDs several women mentioned lack of privacy as a source of embarrassment due to being exposed and watched by other women and health care workers, and stated that lack of privacy was a reason they would not return to give birth in the hospital. Less than two thirds of surveyed women (61.4%) reported that they had been covered with a cloth or blanket or screen during labor and childbirth. Several women said they felt completely exposed, some saying they had been left “half naked” during labor.

*When I went into the emergency room, the doctor who treated me told me to take off my clothes in front of family members. I took off all my clothes in front of my family because they had come with me. My mom, my dad, my husband, my grandma and my sisters-in-law were there. I felt very embarrassed because they did everything in front of them. The truth is they made me feel very bad and I felt ashamed in front of my family. The ones who did that were the nurses, who were Ixil people. The truth is that experience marked me. I don’t want to go back to the hospital again…”* (IDI with a woman who had previously delivered in a facility, Nebaj)

During IDIs many women reported to dislike the hospital gowns, because they are made with a thin fabric and are relatively short providing coverage only to the knees (compared to the traditional *corte* garment worn by indigenous women that covers women down to their ankles). Women also commonly complained about gowns being in bad condition. Many women associated the hospital with physical coldness and found it difficult to keep their bodies warm (the gowns were too thin, only one blanket is usually provided) and described the food as cold; traditionally women are offered hot *atoles* and broths after birth.^1^

Approximately one third of surveyed women (34.3%) reported that health workers explained what was happening and what to expect. Slightly more than half of surveyed women (60.0%) reported that providers explained the reason for performing examinations and procedures or administering medication. Approximately two thirds of women (64.3%) felt that they could ask questions about their care. During qualitative interviews, several women raised the challenge of not being able to communicate with health workers in their language, and cited lack of Spanish as a common reason that health care workers mock women (a form of verbal abuse). By contrast, 94.7% of surveyed women in the three hospitals noted that health care workers spoke to them “in a language that they could speak”.

Over two thirds of surveyed women (70.7%) reported that health workers asked for their consent before conducting exams, procedures or administering medicine. Of the 54 women participating in community-based IDI’s, several women complained about receiving multiple digital vaginal examinations (often from multiple persons) without being asked for consent. Twenty-one of the 54 interviewed women reported undergoing an episiotomy and only two of these 21 women reported being informed (not consented) prior to the episiotomy. Many women complained of experiencing significant pain due to an episiotomy or suturing after birth without enough (or any) anesthesia and stated that this was a reason they did not want to return to the hospital.

*They cut me with scissors from below and they told me that was normal. With the pain I had after that I couldn’t sit up, I was in a lot of pain […] they started stitching me up and they didn’t use anesthesia for that. I felt the worst pain in the world. That’s why I don’t want to go back there, because I felt when they were sticking the needle in and when they pulled it out. I did tell a doctor there I was in pain and he’d say: “Just a bit longer, woman, deal with it”. But, oh God, the pain! I felt everything they did to me, the pain of the needle every time it went in and when they pulled the thread.... At least they should have used anesthesia, right?* (IDI with a woman who had previously delivered in a facility, Santa Cruz)

Less than one fifth of women averaged across the 3 facilities reported that they were “allowed to have a birth companion” during labor ranging from 69.0% in Hospital #1 to 2.8% in Hospital #3 and 1.5% in Hospital #2. As seen in Table 2 the numbers were even lower for women allowed to have a companion during *birth.*

Most of the women interviewed in the community perceived that not all women are treated in the same way during childbirth in the hospital. Many women stated that women who are friends or relatives of health care providers or who are *ladina* (not indigenous) tend to be treated better. During interviews, many women stated that women who do not speak Spanish or who scream and complain about pain receive the worst care and several women noted that poorer women and older women are not treated well.

*The doctor’s friends receive better care, they are treated really well. If they don’t speak Spanish, if they don’t speak clearly or if the people from the villages don’t speak Spanish... I’ve seen how they make fun of them for not speaking Spanish or not expressing themselves well. I’ve seen it. They are told to speak properly, and they (the providers) start laughing. They say they didn’t hear, and they laugh when they repeat themselves. All sorts of people arrive at the hospital, but indigenous people aren’t treated well. That is disrespect, we’ve talked about that with a friend of mine. They don’t treat poor people the same way either, they keep them waiting, I’ve seen. They tell them to go to the social worker to get medications, but they don’t give them to them and keep them waiting.* (IDI with a woman who had previously delivered in a facility, Uspantan)

Of the 64 women (45.7% of the 140 surveyed) reporting any form of mistreatment (any negative care item in Table 2), 17.2% said that their belonging to an indigenous ethnic group was the main reason for mistreatment, except in Hospital #1, where women said they thought that the number of children they had was the main reason for their mistreatment (number of children was the second most cited reason for mistreatment among surveyed women in the other two hospitals). The quantitative survey did not include an option for lack of Spanish as a response to this question although 3.1% said they thought they were mistreated because they did not speak or understand Spanish. Other factors cited by women as perceived reasons for mistreatment included their level of education (3.1%), their complaining about pain (3.1%) and their age (1.5%).

### Health system factors

Approximately one-third of surveyed women (33%) thought that delivery rooms were “very crowded” and one quarter of women (26%) thought there were insufficient personnel to provide care. More than half of surveyed women (55%) reported not having access to a private toilet after giving birth, and about 12% reported not having access to a shower or bath after delivery. During interviews, some women identified health system deficits as contributors to poor quality care, including lack of staff, long health worker shifts and scarce resources.

*Sometimes the hospital is not to blame but the government. You go to the hospital, and what are they going to do if there are no drugs? Some doctors may want to give them to you, because there are really poor people who really need them, but they just don’t have them. So, I believe doctors are not to blame, but I think maybe they don’t ask for these things and the government doesn’t even know.* (IDI with a women who had previously delivered in a facility, Santa Cruz)

### Health worker-reported witnessing of mistreatment of women during childbirth

Results from the survey of 31 health workers mirror many of the client survey results with respect to the mistreatment of women during labor and childbirth. Nearly one-half (45.0%) of surveyed health workers reported ever witnessing a woman in labor or childbirth being verbally abused by a facility health care worker (shouted at, screamed at, insulted or scolded). Few health workers (3.0%) reported ever witnessing a woman being physically abused by a facility health worker (slapped, pinched or punched). None of the surveyed healthcare workers reported ever witnessing a woman being sexually assaulted or harassed by a facility health worker, and 16% of surveyed health workers (all nurses) reported “ever doing anything that made me feel I had disrespected or abused a woman in labor or childbirth”.

### Women’s and providers’ definition of respectful maternity care

During community-based IDIs, women struggled to describe what they consider “respectful care during childbirth”; however, women were very clear about what is NOT respectful care, including mocking women and leaving women exposed without clothing. The characteristic of RMC most frequently mentioned by women during IDIs was “*talking gently and not shouting*”. Other characteristics of RMC commonly mentioned by interviewed women included speaking to women in their maternal language (K’iche’ or Ixil); not ridiculing non-Spanish speakers; protecting women’s modesty and not forcing them to undress in front of family members; accompanying and showing empathy to women during labor; asking what position a woman would like to give birth in; allowing a woman to have someone to accompany her in birth [companion]; and not being left unattended and on her own. The characteristics of RMC described by health care workers (both *comadronas* and facility-based providers) were similar to women’s descriptions of RMC and mostly centered around effective communication (speaking politely, giving explanations, and respecting women’s culture and customs). In addition, *comadronas* described a broader concept of RMC that includes showing respect to both the woman and her family, fulfilling specific rituals, and providing empathy, accompaniment and emotional support.

### Provider’s attitudes about caring for women during childbirth

Health care workers surveyed in the three hospitals were asked whether they agreed or disagreed with a range of statements to elicit their beliefs and attitudes about caring for women during labor and childbirth. Approximately one quarter of surveyed providers (25.8%) agreed with the statement that “it is not always necessary to obtain consent when conducting an examination” and close to one half of providers (41.9%) agreed that “women with lower levels of education may create more problems in childbirth”. Most providers (80.6%) agreed with the statement that “women who are not treated respectfully are less likely to come back to the facility”.

### Qualitative interviews with facility health workers

During qualitative interviews (*n* = 12), most administrators and health workers described maternity care as generally respectful in their facility. They cited a range of factors that may contribute to mistreatment of women in childbirth including excessive workloads (most commonly cited), inadequate infrastructure, overcrowding of facilities, too many patient visitors, health worker fatigue, lack of patient cooperation, excessive complaining by patients, and lack of understanding of hospital routines among women from rural areas and women who do not speak Spanish.

*The main reason would be the language, although this isn’t exactly a barrier to providing respectful care […] We’ve always treated them well, but now people won’t forgive anything. For example, if someone treats someone bad or something, they’ll make accusations right away. Or they’d said: “Look, I want to file a complaint”. […] I don’t think this is due to disrespect, but rather that they don’t understand a particular situation, because of the language or something else, but there are no actual instances of disrespect as such.* (IDI, nurse, Santa Cruz)

Several facility health workers complained about the rude behavior and lack of empathy of some co-workers toward women, noting how hard it is to remove rude personnel from their position even after a formal complaint. Several health care workers also noted that equipment and personnel have not kept up with the increasing demand for institutional births and attributed the demands of providing urgent care for women with obstetric complications as a reason that they are not always able to explain to women what they are doing.

*Unfortunately, our patients arrive in complicated conditions and at the last moment. So, many times, we have to act fast […] So, this affects the speed we do things with and the tone in which we speak to them. If we need to move a patient from one bed to another, we’re a bit firmer when we ask her to let us move her. “Ma’am, let’s move over there, we’re going to move you here”. We don’t have time for: “Excuse me, ma’am, we’re going to move you, if you feel any discomfort, please tell me”. This is obviously due to the urgency of our job. We’re seeing how to improve triage, since we don’t have a person in charge of the classification for the emergency service. So, the security guard has to make this classification.* (IDI, physician, Santa Cruz)

### Association between women’s background characteristics and positive and negative attributes of care

The association between women’s background characteristics and positive and negative attributes of care scores was explored. As shown in Table 3, after adjusting for marital status, parity, and education, an association was found between a positive care score and language spoken at home, where women who spoke Ixil or K’iche at home had a lower likelihood of a positive score aRR: 0.83 (0.72-0.94) compared to women who did not speak Ixil or K’iche at home. After adjusting for marital status, parity, and education, an association was found between negative care score and language spoken at home, where women who spoke Ixil and K’iche at home had a higher likelihood of a negative score (aRR: 2.28 95% CI: (1.30–3.98) when compared to women who did not speak these languages at home. To summarize, speaking Ixil or K’iche at home was associated with a higher likelihood of being treated negatively.

**Table 3:** Association between women’s background characteristics and positive and negative attributes of care scores

**Characteristics**	**RR (95% CI)**	***p*** **value**	**Adjusted RR (95% CI)**	***p*** **value**
**Positive attributes of care score**				
Woman’s age category (ref = 15–19)				
20–24	0.93 (0.80–1.08)	0.36	0.92 (0.79–1.06)	0.23
25–29	0.91 (0.78–1.07)	0.26	0.92 (0.78–1.09)	0.36
30–34	1.06 (0.88–1.28)	0.54	1.14 (0.95–1.39)	0.16
35 +	0.89 (0.73–1.09)	0.26	0.89 (0.73–1.09)	0.28
Marital status (ref = single)				
Married/cohabiting	0.99 (0.81–1.21)	0.92	–	**–**
Parity (ref = primiparous)			–	**–**
2–3 births	1.02 (0.90–1.14)	0.78	–	**–**
4 + births	1.11 (0.95–1.30)	0.17	–	**–**
Speaks Ixil or K’iche at home (ref = no)				
Yes	0.87 (0.76–0.98)	**0.02**	0.83 (0.72–0.94)	**0.004**
Hospital (ref = Hospital #1)				
Hospital #2	0.84 (0.73–0.95)	**0.007**	0.81 (0.71–0.93)	**0.002**
Hospital #3	0.98 (0.85–1.14)	0.82	0.94 (0.81–1.10)	0.45
Education (ref = None)				
Primary	0.96 (0.80–1.16)	0.70	–	**–**
Secondary +	0.95 (0.79–1.15)	0.62	–	**–**
Wealth quintile (ref = quintile 1)				
Quintile 2	0.90 (0.77–1.05)	0.18	–	**–**
Quintile 3	0.92 (0.80–1.05)	0.23	–	**–**
**Negative attributes of care score**				
Woman’s age category (ref = 15–19)				
20–24	1.35 (0.83–2.22)	0.22	–	**–**
25–29	0.98 (0.54–1.77)	0.94	–	**–**
30–34	0.73 (0.34–1.58)	0.43	–	**–**
35 +	0.90 (0.44–1.83)	0.77	–	**–**
Marital status (ref = never married)				
Married/cohabiting	0.54.(0.32–0.91)	**0.02**	0.645 (0.37–1.12)	0.12
Parity (ref = primiparous)				
2–3 births	0.61 (0.40–0.92)	**0.02**	0.72 (0.47–1.12)	0.14
4 + births	0.71 (0.41–1.21)	0.21	0.67 (0.37–1.21)	0.18
Speaks Ixil or K’iche at home (ref = no)				
Yes	2.15 (1.25- 3.71)	0.005	2.28 (1.30–3.98)	**0.004**
Hospital (ref = Hospital #1)				
Hospital #2	1.36 (0.87–2.14)	0.18	–	**–**
Hospital #3	0.88 (0.50–1.55)	0.67	–	**–**
Education (ref = Primary)				
Secondary	0.56 (0.32–0.97)	**0.04**	0.55 (0.32–0.97)	**0.04**
Tertiary	0.79 (0.45–1.38)	0.40	0.83 (0.46–1.50)	0.54
Wealth quintile (ref = quintile 1)				
Quintile 2	1.03 (.062–1.70)	0.91		
Quintile 3	0.84 (0.52–1.33)	0.45		

*RR* relative risk, *aRR *adjusted relative risk

### Providers’ experience of mistreatment

Nearly every surveyed provider (93.5%) reported that she or he or a co-worker had ever experienced verbal abuse (being screamed at, insulted or threatened) by a patient or family member (58.0%) or by a colleague or supervisor (35.4%). Over one fifth of providers (22.5%) reported that she or he or a co-worker had ever been slapped, pinched or punched by a patient or patient’s family member (12.9%) or by a co-worker (9.6%). Ten out of 31 (32.3%) providers reported ever being threatened with harm by a patient or family member. Almost one-fifth of respondents (19.4%) reported ever being threatened by a supervisor that their career would be harmed.

### Provider survey burnout results

The provider survey adapted several items from the Maslach Burnout Inventory (BMI), a widely used instrument to measure occupational burnout at the individual level that incorporates 3 subscales of constructs associated with burnout: emotional exhaustion, depersonalization and personal achievement [19]. As shown in Table 4, with respect to the survey personal achievement subscale items, most providers reported feeling that they were positively influencing patients’ lives often (90.3%) or some of the time (6.5%) and feeling energized or excited about their job often (93.5%) or some of the time (6.5%). Conversely, with respect to survey depersonalization subscale items, one quarter (25.7%) and one third (35.5%) of providers respectively reported worrying that their job was making them less caring often or some of the time and 16.2% and 51.6% providers, respectively, reported that they felt that patients blamed them for things they could not control often or some of the time. With respect to survey emotional exhaustion subscale items, most providers reported feeling emotionally drained often (22.5%) or some of the time (67.7%) and feeling stressed out about their work often (12.9%) or some of the time (64.5%).

**Table 4:** Assessment of provider “burn-out” (*N* = 31 surveyed providers)

Variable	**Total** ***N* = 31** n (%)
**How often do you feel happy with your work providing childbirth care?**	
Often	30 (96.7)
Some of the time	1 (3.2)
Never	0 (0.0)
**How often do you feel emotionally drained from your work?**	
Often	7(22.5)
Some of the time	21 (67.7)
Never	3 (9.6)
**I feel that I take care of certain patients/mothers impersonally, as if they were objects**	
Often	1(3.2)
Some of the time	0 (0.0)
Never	30 (96.7)
**How often do you feel that you are positively influencing your patients’ lives?**	
Often	28 (90.3)
Some of the time	2 (6.45)
Never	1 (3.2)
**How often do you worry that this job is making you less caring?**	
Often	8 (25.8)
Some of the time	11 (35.5)
Never	12 (38.7)
**How often do you feel energized or excited by your job?**	
Often	29 (93.5)
Some of the time	2 (6.5)
Never	0 (0.0)
**How often do you feel frustrated by your job?**	
Often	3 (9.7)
Some of the time	11 (35.5)
Never	17 (54.8)
**How often do you feel like you do not really care what happens to some patients?**	
Often	1 (3.2)
Some of the time	1 (3.2)
Never	29 (93.5)
**How often do you feel stressed by your work?**	
Often	4 (12.9)
Some of the time	20 (64.5)
Never	7 (22.5)
**How often do you feel satisfied after working closely with your patients?**	
Often	31 (100.0)
Some of the time	0 (0.0)
Never	0(0.0)
**How often do you feel blamed by your patients for things you cannot control?**	
Often	5 (16.1)
Some of the time	16 (51.6)
Never	10 (32.3)

## Discussion

This mixed method assessment of women’s experience of facility childbirth care and of health workers’ observations, attitudes and experience of providing care in three hospitals in the Quiché department of Guatemala paints a mixed picture of how women are treated in childbirth and illuminates provider, health system and client factors that influence women’s experience of childbirth.

Both women and health workers identified strong health worker communication as an essential element of RMC. However, as reported by women in our study, health worker communication was often poor (e.g. only one third of surveyed women reported that providers “explained what was happening and what to expect”). This result is consistent with findings from a three-country assessment of person-centered maternity care (PCMC) using a validated 30-item scale that demonstrated the lowest results for the communication sub-scale among three sub-scales (communication, emotional support, respect and dignity) [5].

Consistent with studies from multiple countries, verbal abuse was the form of client mistreatment most commonly reported by both women and providers, with one-fifth of surveyed women reporting verbal abuse by providers. Similarly, a 2016 qualitative study in Guatemala found that yelling was the most common form of mistreatment of women in childbirth based on focus groups in 13 municipalities [8]. Consistent with other studies, women reported higher verbal abuse during community-based interviews and focus group discussions than during facility-based surveys [17]. Women may be more likely to describe negative experiences of facility childbirth away from the facility and after they have had time to process their childbirth experience. The higher rate of provider-reported witnessing of verbal abuse of women (50.0%) than women-reported experience of verbal abuse (21.0%) in the hospital surveys is consistent with findings from a study of providers’ and women’s perspectives on PCMC in Kenya as well as a study in Kenya that included observation of childbirth [17, 20]. Provider surveys (as a complement to surveys of postpartum women) may represent a more feasible method than observation for regularly monitoring mistreatment in programs working to improve RMC and reduce verbal abuse (given women’s low expectations of RMC in many settings).

Women in our study reported frequent painful digital examinations by multiple providers and high rates of episiotomies and laceration repairs, usually without explanation or consent, and sometimes without anesthesia. This last finding is not unique to Guatemala; a qualitative study by Dullo and colleagues (2019) in Ethiopia also found that providers sometimes performed painful episiotomy without anesthesia [21]. Episiotomies, including suturing after birth without informed consent and without anesthesia, may have significant repercussions on a woman’s reproductive and sexual life and mental health [22]. In our study, women cited episiotomies without anesthesia and cesarean sections as reasons for choosing not to give birth in a facility. It is not possible to comment on the proportion of cesarean deliveries that may or may not have been medically indicated among women surveyed in the three referral hospitals, as information on referrals and obstetric complications was not collected for individual women. Nonetheless, the high rate of cesarean delivery among surveyed women in the three hospitals (ranging from 30.6% to 67.7%) likely contributes to the fear of institutional childbirth among many women [9].

Multiple studies have demonstrated that emotional support and companionship during labor and delivery increase the likelihood of vaginal birth (hence reducing the need for cesarean section or assisted delivery), shortens the duration of labor, and improves women’s satisfaction with care [23]. When asked to describe RMC, several women mentioned, “talking gently and not shouting” and “accompanying and showing empathy to women in labor”. Nevertheless, over one fifth of surveyed women reported verbal abuse and less than one in five surveyed women reported being “allowed to have a birth companion” during labor, with even lower numbers of women reporting a companion during birth. This finding is consistent with several studies from countries outside of Guatemala [24]. A study by Peca and colleagues that modeled the relationship between women’s perceptions and future intention to use institutional maternity care in the Western Highlands of Guatemala demonstrated that perceived need for facility-based childbirth services and satisfaction with the last childbirth experience, either at home or in the community, were key factors that influenced intention to give birth in a health institution in the future [7]. Further investigation to understand women’s preferences for emotional support during labor and delivery, including companionship, and the underlying reasons why women are not offered the emotional support they desire, can help inform local interventions to strengthen emotional support of women during childbirth in facilities and potentially increase their utilization of childbirth services.

Many interviewed women and *comadronas* highlighted the importance of Mayan cultural values and childbirth customs. When asked to describe RMC, women mentioned being cared for in their maternal language (K’iche’ and Ixil), having their modesty respected, being asked what position they would like to give birth in, and maintaining physical warmth in line with the importance of hot and cold elements in Mayan cosmology. In interviews, *comadronas* mentioned the importance of supporting family and cultural rituals for women’s and families’ experience of RMC. Many of the municipalities in the Quiché department are at a high elevation and are frequently damp and chilly and many women described feeling cold and exposed when forced to wear thin gowns in unheated facilities. This concept of coldness is also linked to the rejection of hospital food by some women if they are not offered the traditional hot *atoles,* teas or broths. These findings are consistent with the results from a white paper (2017) in which *comadronas* (traditional midwives) and some women reported that hospital providers were impatient and ignorant of their ancestral medicine system, often requiring women to give birth in a hospital gown and sometimes to take a cold shower at admission [25]. Such practices may decrease women’s utilization of or satisfaction with facility-based childbirth services [25].

During IDIs and FGDs, many women described higher rates of verbal abuse directed at certain groups of women, particularly women who do not speak Spanish well or at all, women who complain a lot, who are indigenous, who have many or no children, and who are poor or uneducated. Regression analysis of the client survey results demonstrated a lower likelihood of *positive* care and a higher likelihood of *negative* care among women who spoke Ixil or K’iche’ as their primary language at home. Women who were unmarried or not cohabitating and women who were not educated to a secondary level were also more likely to report *negative* care attributes. Qualitative interviews with women reinforce the regression analysis results, with many women describing the mocking and humiliation by providers of women who do not speak Spanish well. Provider survey results suggest that provider attitudes influence the discrimination against certain women described by women. Nearly one half of surveyed providers agreed with the statement that women with lower levels of education may cause more problems and one fifth of providers agreed that it is reasonable to detain women who are unable to pay fees for maternity services. These findings are consistent with other studies which found that social discrimination plays a prominent role in indigenous people’s experience of health care services in Guatemala [8]. Providers’ deeply held, often unconscious biases based on ethnicity, class, and gender likely contribute to discrimination against certain groups of women [10]. The deep power imbalance between *ladino* and indigenous persons, rooted in Guatemala's colonial history, is an important contributor to the increased vulnerability of indigenous women as reflected by the results of the regression analysis.

Both providers and clients agreed that structural issues hinder the provision of quality, respectful care, in particular an excessive workload due to the lack of hospital personnel and the lack of enabling infrastructure (e.g. lack of curtains to ensure visual privacy; women’s lack of access to a private toilet). These findings align with a 2015 systematic review by Bohren et al. which found that health system factors, such as a lack of privacy or staffing shortages, can be experienced directly by women as mistreatment [3]. Our study’s findings are consistent with the global literature that suggests mistreatment is not merely an interpersonal problem, but is also driven by health system inadequacies that affect clients and providers alike [10, 26, 27].

Provider survey results demonstrate high levels of verbal abuse of health workers perpetrated by clients, client family members and colleagues and mixed results with respect to provider-reported burnout symptoms. While it is not possible to comment on burnout results at the individual provider level in our study, the high proportion of providers reporting emotional exhaustion and depersonalization some or most of the time is concerning, although counterbalanced by the many providers who reported feelings of positive personal achievement in their work. The greater than 90% of providers in our study who reported verbal abuse against themselves or a colleague mirrors findings from a study in Addis Ababa, Ethiopia that found that over half of surveyed maternity care providers (nurses, midwives, doctors) reported being disrespected or abused in the work place by a patient, family member or colleague [28]. The 2016 global consultation, “Midwives’ Voices, Midwives’ Realities,”, that enumerates many of the challenges described by providers in our study, asserts that the ‘working environment’ must become a ‘caring environment’ that is responsive to the needs of both healthcare providers and the women they care for [25].

In collaboration with MSPAS, the MCSP program convened a series of community meetings with local community and hospital stakeholders to discuss assessment findings and begin to explore local interventions to address identified barriers to and facilitators of RMC. Preliminary findings from these meetings suggest an increased shared understanding of women’s perspectives and needs and understanding of hospital maternity care processes and facility limitations as well as shared appreciation for the value of joint dialogue and action among community members and facility health workers to improve respectful maternity care services.

### Limitations

There are several limitations to this study. The assessment was conducted in only three hospitals and these facilities may not be representative of all hospitals or other types of facilities in the study area. The inability to guarantee auditory privacy during client surveys in one of the three hospitals may have influenced women’s responses. Due to the small sample size, it is not possible to generalize the health worker survey results to the Quiché department or beyond. The lack of background demographic information for women participating in the community based IDIs and FGDs is another limitation of the study since it is not possible to assess whether differences in quantitative and qualitative results may have been due in part to differences in the background characteristics of women participating in the facility questionnaire versus the IDIs and FGDs. Although the provider and client questionnaires were pre-tested and incorporated many items from questionnaires or scales validated in other settings, a limitation of the study is that the client and provider questionnaires were not validated in the specific study setting.

## Conclusions

This study contributes to the understanding of women’s experience of hospital maternity care and the factors that influence this care in the Quiché Department of Guatemala, and adds to the global RMC literature by combining qualitative and quantitative methods to triangulate women’s and health workers’ experience of and perceptions of RMC in facilities. Assessment findings highlight many positive assets on which to build in this setting and, also, illuminate many factors that contribute to mistreatment of women and providers and must be addressed to improve respectful maternity care.

## Data Availability

The datasets generated and analyzed during the study are not currently publicly available due to limitations of ethical approval involving the patient data and anonymity but are available from the corresponding author on reasonable request.

## Abbreviations

BMI: Maslach Burnout Inventory
FGD: Focus group discussion
KII: Key informant interview
PCMC: Person-centered maternity care
RMC: Respectful maternity care
WHO: World Health Organization

## Footnotes

1 In Mayan cosmology, giving birth is a “hot” process as the body opens up and maintaining heat is viewed as an essential aspect of recovery for the woman and the infant; allowing a woman or infant to become “cold” is thought to jeopardize well-being.
